# Risk factors associated with surgical site infections following joint replacement surgery: a narrative review

**DOI:** 10.1186/s42836-022-00113-y

**Published:** 2022-05-01

**Authors:** Tao Li, Haining Zhang, Ping Keung Chan, Wing Chiu Fung, Henry Fu, Kwong Yuen Chiu

**Affiliations:** 1 Department of Joint Surgery, The Affiliated Hospital of Qingdao University, Qingdao, China; 2 Department of Orthopaedics &, Traumatology Queen Mary Hospital, The University of Hong Kong, 102 Pok Fu Lam Road, Hong Kong SAR, China

**Keywords:** Surgical site infection, Risk factor, Joint replacement

## Abstract

**Background:**

Surgical site infection following joint replacement surgery is still a significant complication, resulting in repeated surgery, prolonged antibiotic therapy, extended postoperative hospital stay, periprosthetic joint infection, and increased morbidity and mortality. This review discusses the risk factors associated with surgical site infection.

**Related risk factors:**

The patient-related factors include sex, age, body mass index (BMI), obesity, nutritional status, comorbidities, primary diagnosis, living habits, and scores of the American Society of Anesthesiologists physical status classification system, *etc.* Surgery-related factors involve preoperative skin preparation, prolonged duration of surgery, one-stage bilateral joint replacement surgery, blood loss, glove changes, anti-microbial prophylaxis, topical anti-bacterial preparations, wound management, postoperative hematoma, *etc.* Those risk factors are detailed in the review.

**Conclusion:**

Preventive measures must be taken from multiple perspectives to reduce the incidence of surgical site infection after joint replacement surgery.

## Introduction

Surgical site infection (SSI) represents one of the major complications of joint replacement surgery (JRS). It possibly extends postoperative hospital stay, prolongs the antibiotic therapies, and leads to periprosthetic joint infection (PJI). PJI is a devastating and challenging complication that increases morbidity and mortality rates [1]. Although the incidences of PJI range from 1% to 2.4% [2, 3], it may cause significant psychological stress to patients and pose a heavy economic burden to a country [4]. Many orthopedic surgeons are focusing on the potential ways to minimize the occurrence of SSI. Multiple researchers have pointed out that the SSI rate is multi-factorial, including patient susceptibility and environmental and social aspects. In this review, we elaborate on the risk factors associated with post-JRS SSI, with a hope to arouse orthopedists’ attention to SSI and PJI.

### Patient factors

#### Sex and age

Older patients are particularly vulnerable to post-total hip arthroplasty (THA) infection due to low immune resistance and poor nutritional status [5–8]. However, Muilwijk *et al*. [9] revealed no association between age and SSI after primary THA. Baier *et al*.[10] did not find statistically significant differences between the age groups in terms of SSI after total knee arthroplasty (TKA). Many studies demonstrated that male patients had a higher SSI and PJI rate than their female counterparts and the difference was more significant in TKA [11–15].

### Body Mass Index (BMI) and Obesity

Obese patients have relatively higher SSI rates than non-obese patients [6, 14, 16–18]. A BMI ≥ 32 was found to be associated with high risks of SSI [19]. Falagas *et al*. [20] found that adipose tissue played an active role in inflammation and immunity through multiple inflammatory cytokines and immune mediators, making obese patients more susceptible to infection. Therefore, preoperative weight control and dieting are critical to reducing the risk of SSI.

#### Nutritional status

Malnutrition is one of the common risk factors. Pruzansky *et al*. [21] determined albumin or total lymphocyte levels to assess the nutritional status and found that malnutrition was associated with a high incidence of SSI. Rasouli *et* *al*. [22] revealed that low preoperative hemoglobin levels increased the risk of SSI.

### Comorbidities

#### Diabetes mellitus

Diabetes Mellitus is associated with wound complications [6, 23, 24]. Pruzansky *et al*. [21] reported that 20% of patients with SSI had diabetes. In diabetic patients, inadequate perioperative glycemic control is associated with an increased risk of postoperative SSI. HbA1c is a biomarker for diagnosing and treating diabetes, but a cutoff of 7% alone cannot be used for risk stratification [25]. Fructosamine, glycosylated albumin, and 1.5-anhydroglucytol may be more sensitive measures because HbA1c often produces false findings in patients with both chronic kidney disease and anemia [26].

#### Rheumatoid arthritis

Patients with rheumatoid arthritis are prone to SSI because they often receive hormones and immunosuppressants. Many studies have confirmed that rheumatoid arthritis is associated with a high incidence of SSI [16, 27, 28].

#### Asymptomatic bacteriuria

Asymptomatic bacteriuria and urinary tract infection are the two major urinary tract diseases leading to an increased incidence of SSI. In a multicenter cohort study that enrolled 2,497 JRS patients, Sousa *et al*. [29] reported that the PJI rate was significantly higher in the asymptomatic bacteriuria group than in the non-asymptomatic bacteriuria group (odds ratio, 3.23; Cl, 1.67–6.27; *P* = 0.001). In a systematic review and meta-analysis, Gómez-Ochoa *et al*. [30] exhibited that the SSI incidence of the asymptomatic bacteriuria group was higher (2.3% *vs.* 1.1%). Parvizi *et al*. [31] found that urinary tract infection was an independent predictor for PJI. The latest research findings showed preoperative (not postoperative) urinary tract infection increased the risk of superficial wound infection and deep PJI [32].

#### Peripheral vascular disease

Marusic *et al*. [33] reported that early peripheral vascular diseases led to 6.4 times and 3.5 times the risks of SSI after THA and TKA, respectively, due to tissue hypoxia and delayed wound healing. Park *et al*. [34] demonstrated that the post-TKA arterial complications probably led to delayed wound healing, skin necrosis, deep infection and other complications.

#### Chronic skin disease

A 4-year retrospective cohort study that included 2439 patients, suggested that chronic skin disease (*e*.g*g*., atopic dermatitis, psoriasis) was involved in an increased risk of SSI [9]. Kawata *et al*. [10] also revealed that atopic dermatitis was an independent demographic risk factor for SSI after anterior cruciate ligament reconstruction.

#### Revision JRS

Revision JRS carries a higher SSI rate due to the long operative time and the previous scars that affect wound healing. De Jong *et al*. [35] suggested the deep infection rate of hemiarthroplasty was increased following femur neck fractures (OR 15.2, *P* < 0.001). Everhart *et al*. [28] reported that the infection rate of revision shoulder arthroplasty was 9.1%. Pugely *et al*. [36] demonstrated that SSI rates of revision JRS was high, especially in hip revision arthroplasty.

#### Other factors

Femoral head osteonecrosis, osteoarthritis, post-traumatic arthritis, hip dysplasia may not be associated with SSI [14].

### Living habits

#### Smoking

Many studies showed that smoking increased the incidence of postoperative SSI. In a systematic review of primary JRS patients, Bedard *et al*. [37] showed smokers had significantly higher risks of wound complications and PJI than non-smokers in both current and former smoking settings. Liang *et al*. [19] indicated that smoking (OR, 4.2; 95% CI, 2.1–6.4) was an independent risk factor associated with SSI.

#### Alcohol abuse

Few articles reported on the relationship between alcohol abuse and SSI. Wu *et al*. [6] and Poultsides *et al*. [12] demonstrated that alcohol abuse carried a 1.57- to 2.95-fold higher risk of PJI. Excessive alcohol consumption (45 g/day for men and 30 g/day for women) is commonly found in patients with an infected THA due to impaired liver function and a weakened immune system [7].

#### American Society of Anesthesiologists (ASA) Score

Anesthesiologists often use the ASA score to assess patients’ overall preoperative health condition. Paryavi *et al*. [38] showed that ASA class 3 or higher predicted infection with an odds ratio of 2.87. Namba *et al*. [14] found ASA classes 1 and 2 were more closely associated with lower SSI rates than the higher ASA classes (0.34% *vs.* 0.80%). Yang* et al*. [39] demonstrated that the adjusted odds ratios in univariate and multivariate analyses for ASA score were 1.77 and 3.36, respectively. They found that an ASA class ≥ 3 was an independent risk factor for SSI.

### Surgery-related factors

#### Preoperative skin preparation

The optimal disinfectant used for preoperative skin preparation remains controversial. Zywiel *et al*. [40] found chlorhexidine gluconate-impregnated cloth in the night and morning appeared to reduce the SSI incidence effectively, compared to nosocomial skin preparation alone. In a systematic review and meta-analysis, Cai [41] displayed that chlorhexidine used for preoperative skin preparation could significantly reduce the risk of SSI (*RD* = –0.02, *P* < 0.001). An alcohol-based solution of chlorhexidine is also recommended as the first choice by the National Institute for Health and Care Excellence (UK) [42]. However, different opinions exist. Swenson *et al*. [43] showed that chlorhexidine (8.2%) was associated with a significantly higher postoperative infection rate than povidone iodine and iodine polyacrylate. Carroll *et al*. [16] revealed that skin preparation using chlorhexidine (0.5%) in alcohol (70%) carried a five-fold increased risk of superficial wound complications, compared to iodine (1%) in alcohol (70%), particularly a 13-fold increased risk in THA. In a cluster randomized controlled trial, Peel *et al*. [44] also pointed out that iodine-alcohol achieved greater efficacy than chlorhexidine-alcohol. Letzelter *et al*. [45] demonstrated that the combination of the two disinfectants worked better, particularly in JRS.

### Prolonged surgery and one-stage bilateral JRS

Prolonged operations increase the time of wound exposure, likelihood of excessive tissue stripping, blood loss, and duration of anaesthesia. All those factors increase the risk of infection. Teo *et al*. [46] revealed that protracted operative time was associated with a high rate of SSI in unilateral TKA (90.5 ± 28.2 min *vs.* 72.2 +± 2 0.3 min; *P* = 0.03). Sheth *et al*. [47] reviewed 17,342 primary unilateral TKAs and THAs and found that the incidences of SSI and PJI were significantly higher if the operative time lasted more than 90 min (2.1% and 1.4%, respectively), compared to the operative time between 60 and 90 min (1.1% and 0.7%) and the time less than 60 min (0.9% and 0.7%, respectively; *P* < 0.01). In a retrospective multicenter study, Liu *et al*. [8] demonstrated the operative time longer than 107 min was significantly associated with a high rate of wound infection (OR, 2.18, *P* = 0.001). Theoretically, one-stage bilateral JRS increases the operative time, the amount of bleeding and the probability of blood transfusion. However, several studies failed to show differences between simultaneous and unilateral or staged bilateral TKA and THA in infection rate [48–52].

#### Glove change

During surgery, surgical gloves may be contaminated, especially in prolonged operations. Glove change reduces the risk of SSI. Kim *et al*. [53] suggested that surgeons should change their gloves at an interval less than one hour. In addition, they should change their gloves after draping, before handling the implant, and if visible perforations are seen.

#### Antibiotic prophylaxis

The use of intravenous antibiotics is the most important measure to decrease wound infection. Siddiqi *et al*. [54] performed a systematic review and meta-analysis based on 31 studies involving 51,627 patients. They demonstrated that the antibiotic prophylaxis was effective in preventing post-JRS infection, but continous use for more than 24 h had no benefit. Christensen *et al*. [55] retrospectively reviewed 6,080 patients and revealed that single-dose of prophylactic antibiotics was not associated with increased acute PJI and short-term complications, compared to a 24-h antibiotic regimen. Some surgeons reported similar results [56, 57]. Patient-reported allergy to penicillin was most likely to be prescribed with clindamycin (1.1 *vs.* 80.7%; *P* < 0.05) or vancomycin (4.0 *vs.* 12.4%; *P* < 0.05) rather than cefazolin (94.9 *vs.* 6.9%; *P* < 0.05) [58]. However, Zastrow *et al*. [59] found that antibiotic prophylaxis regimens (vancomycin, clindamycin or combined regimen with cefazolin) were associated with an increased risk of SSI in THA and TKA, compared to cefazolin alone. Cefazolin underdosing was associated with a high rate of post-JRS SSI. Morris *et al*. [60] suggested that weight-based dosing of cefazolin (body weight ≥ 80 kg, giving 2 g/d; ≥ 120 kg, giving ≥ 3 g/d) should be used in THA and TKA.

#### Local antibiotic use

Antibiotics alone or in combination with certain substances are placed in the joint cavity to attain antibacterial effect. In addition to systemic antibiotic prophylaxis, local administration of gentamicin-collagen sponges reduces SSI incidence in elderly patients undergoing hemiarthroplasty [61]. Peng* et al*. [62] conducted a meta-analysis and found that local use of vancomycin powder significantly decreased the SSI rate after primary JRS but did not change the bacterial spectrum involved. Buchalter *et al*. [63] reported that vancomycin powder combined with diluted povidone-iodine lavage was associated with a decreased incidence of PJI after primary TKA.

#### Wound management

The need to drain is always a controversial issue. In a systematic review on SSI, Resende *et al*. [15] did not find a statistically significant difference in SSI rate between the patients with and without drainage. Doman *et al*. [64] suggested that use of a closed incision negative pressure wound therapy decreased wound complications, compared to closed silver-impregnated dressing (6.9% *vs.* 16.2%, *P* = 0.031). They suggested that a negative pressure wound therapy be used in high-risk patients, especially in patients undergoing non-aspirin anticoagulant therapy. In a prospective randomized controlled study, Kuo *et al*. [65] demonstrated that the incidence of superficial SSI was significantly lower in the Aquacel Ag Surgical dressing group (0.8%, 95% CI: 0.00–2.48) than in the control group (8.3%, 95% CI: 3.32–13.3; *P* = 0.01). Some surgeons suggested that antibiotic-coated sutures be used to decrease SSI, but offered no strong supporting evidence [66, 67].

#### Postoperative hematoma

The chemical thromboprophylaxis used in JRS to reduce deep venous thrombosis and pulmonary thrombosis may cause hematoma. Hematoma is a suitable culture medium for bacterial reproduction and growth. De Jong *et al*. [35] showed that postoperative hematoma was associated with an increased risk of deep infection after hemiarthroplasty. Saleh *et al*. [68] found that hematoma formation (*OR* = 11.8; *P* < 0.05) was a significant predictor of superficial SSI in TKA and THA.

#### Doctors, hospitals, and others

Preoperative *Staphylococcus aureus* screening and decolonization are helpful to reduce bacteria colonization, transmission, and surgical site infection. Saleh *et al*. [69] found methicillin-resistant *Staphylococcus aureus* undergoing the selective treatment showed confirmed eradication, but a significantly increased risk of SSI remains in THA and TKA. This evidence suggests that preoperative bacterial screening and decolonization should be conducted. Weiser and Moucha [70] found that non-colonization of *Staphylococcus aureus* screened in the nasal cavity could potentially reduce the incidence of SSI. Zhu *et al*. [71] revealed that screening and decolonization of nasal *Staphylococcus aureus* significantly reduced the risks of SSI, PJI and superficial infections, compared to the non-decolonization group. The latest systematic review and meta-analysis showed that non-decolonization was associated with a higher risk of *Staphylococcus aureus* infection (*RR* = 2.18 ± 0.41) and other infections (*RR* = 1.70 ± 0.17) in elective JRS [72].

#### Doctor’s operation volume and experience

De Jong *et al*. [35] found that a surgeon’s experience is an independent factor in preventing infection. Surgeons who had performed more JRS had a lower SSI rate. In a prospective cohort study, however, Finkelstein *et al*. [73] did not find a statistically significant difference between the surgeons who performed high-volume operations and those who did low-volume operations (4.3% *vs.* 4.9%, *P* = 0.65).

#### Socioeconomic factors

Wu *et al*. [6] reported that patients living in rural areas had a 2.63-fold increased risk of PJI than urban patients. Rural patients are more likely to experience a delayed diagnosis and treatment due to irregular outpatient visits. Ong *et* *al*. [74] also found that the patients undergoing public assistance for Medicare premium were associated with an increased risk of PJI (OR: 1.34, *P* = 0.005). Patients on low-income government insurance (Medicaid) had a higher incidence of SSI after shoulder arthroplasty during the initial inpatient stay [75]. Compared to Medicare, the patients who primarily had Medicaid were most likely to experience a higher (> 49%) risk of SSI 30 days after TKA [76]. Poor living conditions and nutritional levels, pre-existing comorbidities, non-compliance with medical advice, and failure to seek timely care in a lower socioeconomic environment render the patients more vulnerable to an increased risk of infection [77].

#### Seasonal factors

Kane* et al*.[78] found that incidences of infection were higher in July (4.5%), August (5.4%), and September (4.3%) than in the intermediate periods between summer and autumn months (3.6%) and winter and spring months (1%). For post-TKA infection, the 30-day readmission rate of June (peak) was 30.5% higher than that of December (trough) [75]. For post-THA infection, the readmission rate of July (peak) was 19% higher than that of January (trough). In a retrospective nationwide study, Yamagami *et al*. [79] also showed that summer season was associated with higher rates of post-TKA and post-unicompartmental knee arthroplasty SSI and PJI. It may be attributed to a larger skin bacterial population at higher temperatures and humidity [80]. In addition, the “July effect” (new medical student arrival) was associated with lower quality of wound care and a higher incidence of SSI [77].

#### Prevention of SSI

For invariable factors, such as sex and age, doctors should inform the patients and their family members about the risks. The surgical team should optimize those modifiable factors to decrease the risk of SSI (Table 1).

**Table 1:** A summary of risk factors associated with post-JRS SSI

Non-Modifiable Risk Factor	Modifiable Risk Factor	Prevention of SSI
Patient’s factors		
1. Gender and age	1. BMI and obesity	1. Weight control
2. Primary diagnosis	2. Nutritional status	2. Corresponding dietary support
3. ASA class	3. Comorbidities (diabetes mellitus, rheumatoid arthritis, asymptomatic bacteriuria, peripheral vascular disease, chronic skin disease)	3. Perioperative blood glucose control, optimization of patients’ comorbidities
	4. Living habits (smoking, alcohol abuse)	4. Reduced tobacco and alcohol dependence
Surgery-related factors		
	1. Preoperative skin preparation	1. Iodine-alcohol or chlorhexiodine for preoperative skin prepration
	2. Prolonged duration of surgery and one-stage bilateral JRS	2. Control of the sterility during the operation, shortening of operation time and minimization of the trauma
	3. Glove changes	3. Glove changes
	4. Antibiotic prophylaxis	4. Reasonable antibiotic prophylaxis
	5. Antibacterial preparations for topical use	5. Application of ciNPWT
	6. Wound management	6. The choice of chemical thromboprophylaxis
	7. Postoperative hematoma	
Doctors, hospitals and other factors		
1. Socioeconomic factors	1. Preoperative staphylococcus aureus screening and decolonization	1. Active nasal *Staphylococcus aureus* screening and decolonization
2. Seasonal factors	2. Doctor’s operation volume and experience	2. Strict aseptic dressing change after operation, regular follow-up, and timely management of postoperative complications

*SSI*, Surgical Site Infection

*ASA*, American Society of Anesthesiologists

*BMI*, Body Mass Index

*JRS*, Joint Replacement Surgery

*ciNPWT*, Close Incisional Negative Pressure Wound Therapy

## Conclusions

Preoperatively, the surgeon should fully evaluate patients’ habits, medical history, and health conditions. Some important measures should be taken: (1) quit tobacco and alcohol or even have a cessation program; (2) optimize perioperative levels of blood glucose of diabetic patients; (3) support the patient’s diet to achieve a normal level of hemoglobin and nutritional status; (4) optimize patient’s comorbidities, such as rheumatoid arthritis, peripheral vascular disease, chronic skin disease, *etc*. and (5) perform nasal *Staphylococcus aureus* screening, decolonization, and antibiotic prophylaxis.

Intraoperatively, the surgical team should strictly control the sterility, shorten the operative time, and minimize the trauma as much as possible. Several issues need to be addressed: (1) use Iodine-alcohol for preoperative skin preparation; (2) change gloves after draping, before handling implants or in the presence of perforations; and (3) use a ventilation system with 100-level laminar flow in the operating room.

Postoperatively, the surgeons should strictly keep dressing change aseptic, follow-up patients regularly, and address complications in a timely manner. The patient should be informed to consult surgeons if there are wound redness, swelling, and discomfort in the knee. The patient should be prescribed oral antibiotics to prevent blood-borne infection, if they had dental procedures.

## Data Availability

No applicable

## Abbreviations

SSI: Surgical site infection
JRS: Joint replacement surgery
PJI: Periprosthetic joint infection
THA: Total hip arthroplasty
TKA: Total knee arthroplasty
ASA: American Society of Anesthesiologists
ciNPWT: Closed incision negative pressure wound therapy
BMI: Body Mass Index
